# A systematic review of intellectual and developmental disability curriculum in international pre-graduate health professional education

**DOI:** 10.1186/s12909-023-04259-4

**Published:** 2023-05-11

**Authors:** Lisa Vi, Muhammad Irfan Jiwa, Yona Lunsky, Anupam Thakur

**Affiliations:** 1 University of Toronto, Temerty Faculty of Medicine, Toronto, ON Canada; 2 Campbell Family Mental Health Research Institute, Centre for Addiction and Mental Health, Toronto, ON Canada; 3 Department of Psychiatry, Temerty Faculty of Medicine, Toronto, ON Canada; 4 Azrieli Adult Neurodevelopmental Centre, Centre for Addiction and Mental Health, Toronto, ON Canada

**Keywords:** Intellectual and developmental disability, Developmental disability, Intellectual disability, Education, Medical education, Health professional

## Abstract

**Background:**

Despite the increasing global population of individuals with intellectual and developmental disabilities (IDD), this population remains especially vulnerable to health disparities through several factors such as a lack of access to sufficient medical care and poor determinants of health. To add, numerous studies have shown that healthcare professionals are still insufficiently prepared to support this population of patients. This review synthesizes the literature on current pre-graduate IDD training programs across healthcare professions with the goal of informing the creation of evidence-based curricula.

**Methods:**

Four major databases were searched for current pre-graduate IDD training interventions for healthcare professionals. The Preferred Reporting Items for Systematic Reviews and Meta-Analysis flow diagram and the Best Evidence Medical Educations systematic review guide were used to frame our collection and analysis.

**Results:**

Of the 8601 studies screened, 32 studies were identified, with most studies involving medical students (50%). Of note, 35% of studies were interprofessional. Most interventions utilized multiple pedagogical methods with a majority including clinical experiences (63%) followed by theoretical teaching (59%). Kirkpatrick levels showed 9% were level 0, 6% were level 1, 31% were level 2A, 31% were level 2B, 19% were level 3, 3% were level 4A, and none were level 4B.

**Conclusions:**

There is a paucity of formally evaluated studies in pre-graduate health professional IDD education. As well, there are a lack of longitudinal learning opportunities and integration into formal curriculum. Strengths identified were the use of multimodal approaches to teaching, including interprofessional approaches to optimize team competencies.

**Supplementary Information:**

The online version contains supplementary material available at 10.1186/s12909-023-04259-4.

## Background

Persons with intellectual and developmental disability (IDD) are vulnerable to health disparities. Lack of access to sufficient medical care, poor determinants of health, and exclusion from public health and preventive care are all related to poor health outcomes in this population. Various reports highlight gaps in healthcare for persons with IDD globally [1–3]. Despite the recognition of health inequities, a lack of training to care for patients with IDD has been reported across healthcare professions in medicine [4–9], dentistry [10–12], occupational and physical therapy [13], psychology [14], and nursing [15, 16]. In particular, one study surveyed 714 U.S physicians and found only 40.7% were confident in their ability to provide equal quality care to those with disabilities, and only 56.5% strongly agreed to treat these patients in their practices [17]. Bowen et al., further highlights the need for increased education through their call to action, noting gaps in health education and continuing education curricula in disability competent care [18]. In response to the need for better disability education, a US national consensus on disability competencies for healthcare education was developed which includes 6 competencies, 49 sub-competencies, and 10 principles through collaboration between people with disabilities, disability advocates, family members of people with disabilities, health professionals, and health educators [19]. In addition to these recognized competencies, formal pedagogical structures are needed to equip providers with the skills to effectively care for patients with IDD.

Unfortunately, studies on formal pedagogical structures directed at health providers in IDD care are limited. In a systematic review of post-graduate medical training in intellectual and developmental disabilities a paucity of objectively evaluated research in this area and a potential for specialized, interprofessional, competency-based education programmes were highlighted [4]. While there are post-graduate training programs for those who wish to specialize in IDD care, there lacks consensus on how to train general health professionals on the care of this population. Moreover, with a global shift from institutional to community-based care over the past few decades, patients with IDD depend on the care of general providers to address their health needs [20]. Therefore, IDD education needs to be directed not only at post-graduates but to pre-graduates, prior to specialisation. Currently, there are no known studies that have examined pre-graduate IDD training within broader healthcare professional education.

This study aimed to conduct a systematic review to describe the characteristics and educational outcomes of recent pre-graduate IDD training across various health care professions. The purpose of our review was to synthesize the literature on current pre-graduate IDD training interventions across healthcare professions, with the goal of informing the creation of evidence-based curricula.

## Methods

Our aim was to synthesize the literature on current pre-graduate IDD training interventions for healthcare professionals. To do so, we used the Preferred Reporting Items for Systematic Reviews and Meta-Analysis (PRISMA) flow diagram and the Best Evidence Medical Education (BEME) systematic review guide to frame our collection and analysis.

### Search strategy

The literature was first searched on June 21st 2021, followed by a second search on March 8th, 2023 to provide the latest findings. Ovid and Webofscience were used to search the literature. In particular, Ovid was used to search the Medline, Embase and Psychinfo databases. The search was conducted using subject keywords “or” combinations of student*, trainee*, interprofessional*, and healthprofession* with “or” combinations of developmental disab*, intellectual disab*, ASD, autis*, learning disab*, mental retard*, asperger* with “or” combinations of education*, curricu*, and training. The search was limited to English language, peer-reviewed articles published from 2011 to current, to account for recent and relevant interventions only. Following the initial search, articles of interests’ references were scanned for additional publications.

### Inclusion and exclusion criteria

Studies were included if they were an educational intervention aimed at improving IDD knowledge, skills, self-efficacy, and/or attitudes for any group(s) of pre-graduate health professional trainees. Pre-graduate health professional trainees were defined as trainees within their pre-licensing years of a professional program. Interprofessional interventions that included graduate health professionals or other areas of study were included if they included pre-graduate health professionals as well. No sample size cut-off was employed as the relative paucity of work in this research area was expected. Those studies that included an intervention but had no formal evaluation outcomes, and that evaluated learner’s attitudes, knowledge, skills, and/or self-efficacy without a corresponding intervention were excluded.

### Title and abstract review

**Fig. 1:**
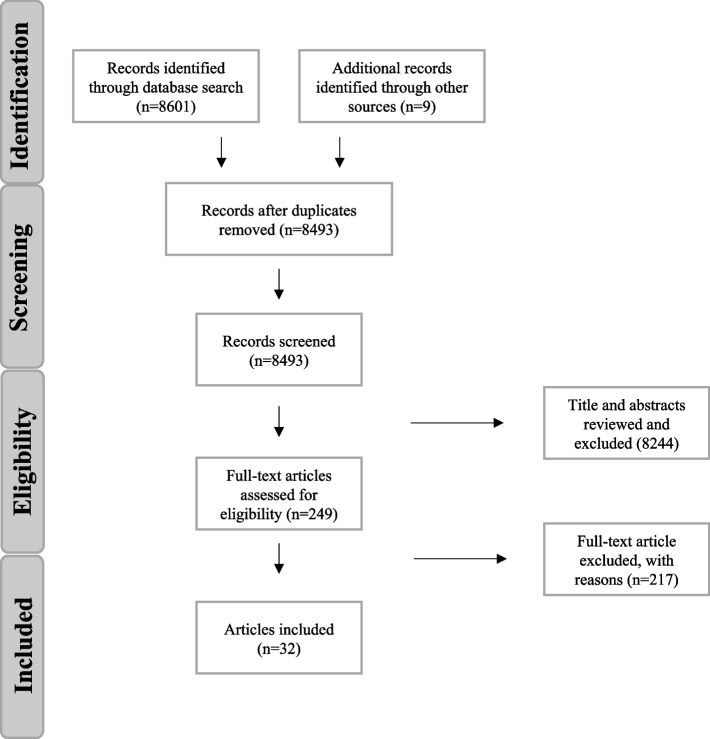
PRISMA flow diagram

The initial database search identified 8601 studies in which, after removing duplicates, reviewing titles and abstracts for relevance yielded 249 articles. After applying inclusion and exclusion criteria, and searching reference lists of significant articles, 32 papers were included in the final review. Study flow is outlined in Fig. 1.

### Full-text review, data extraction, synthesis and analysis

With guidance from all other authors, one author (L.V.) analyzed the core papers, and extracted data from the 32 studies into a table classifying data according to (i) year of publication, (ii) country of origin, (iii) pre-graduate training speciality, (iv) learner level of participants, (v) instructor type, (vi) setting of instruction, (vii) timeline, (viii) pedagogical approach, (ix) focus of content, (x) evaluation method (xi) evaluation outcomes, (xii) Kirkpatrick level, and (xiii) BEME quality of evidence score. Additional file 1: Table S1 summarizes the findings of this analysis.

Instructor data was classified into the following categories: (1) faculty members, (2) non-faculty professionals, (3) patients, parents, or caregivers, (4) senior students, and (5) unclear. The setting of intervention was classified as: (a) specialized clinical setting, (b) non-specialized clinical setting, (c) non-clinical setting, (d) clinical setting (unclear whether specialized or not), and (e) unclear. Next, the timeline of the intervention was classified as: (i) single session, (ii) short-term, less than 1 month, (iii) 1–3 months, and (iv) longitudinal of longer than 3 months. The pedagogical approach was classified as: (1) experiential, sub-stratified into (a) patient/family experiences, (b) clinical, (c) workshops, (2) theoretical, and (3) interprofessional. The focus of content was classified as: (i) perspective/awareness, (ii) medical/clinical knowledge, and (iii) unclear.

Evaluation methods were organized as such: (1) intervention evaluation (participant evaluation of the intervention), (2) participant evaluation (participant evaluation of themselves), (3) learning assessment (assessment of knowledge/skills/perspective gained following intervention), and (4) other. The evaluation outcomes were synthesized from each study, and Kirkpatrick and BEME gradings were applied to all studies. The Kirkpatrick classification was chosen as it has been commonly applied to the evaluation of health professional education programs [21]. The Kirkpatrick classification assesses the effectiveness of education programs according to various levels (level 1-4B). In particular, we used the modified version of the Kirkpatrick model from Steinert et al. which classifies levels as follows: (1) Level 1 – participants reaction(s) to the learning experience, (2) Level 2A – changes in attitudes, (3) Level 2B – Modification of knowledge or skills, (4) Level 3 - change in behaviours, (5) Level 4A – changes in the system/organisational practice, and (6) Level 4B – improvement in students learning/performance as a direct result of intervention [22]. Additionally, the BEME level of evidence grading was used to assess the strength of evaluation outcomes based on grades of: (1) no clear conclusions, (2) ambiguous results, although appearance of a trend, (3) conclusions can probably be based on the findings, (4) results are clear and highly likely to be true, and (5) unequivocal results.

## Results

A summary of study characteristics is available in Tables 1 and 2, with an additional summary of study characteristics displayed in Additional file 1. Specifically, Table 1 provides data on pedagogical methods and evaluation outcomes and Table 2 provides data on intervention delivery, in contrast to the Additional file 1 which organizes the results by study characteristics.

**Table 1:** Review findings with a focus on pedagogical methods and evaluation outcomes

Author, Year of publication, COO	Speciality	Pedagogical Methodology	Focus of Content	Evaluation Method	Evaluation Outcome	Kirkpatrick Level	BEME Scores
[23] **Keisling et al. 2017** **USA**	• Psychology • SLP (speech language pathology) • Audiology • Nutrition • Social work • Nursing	Experiential (patient/family experiences) Theoretical (didactic) Interprofessional	Perspective/Awareness	Participant Evaluation	Improvements in family centered care competencies with comments reflecting a desire for more family centred experiences	3	4
[24] **Weber et al. 2021 ** **USA**	• Audiology • Genetic counselling • Nursing • OT (occupational therapy) • PT (physiotherapy) • Psychology • Social work • SLP • Other non- healthcare professional programs and/or post-graduate trainees^a^	Interprofessional Theoretical (didactic) Experiential (clinical)	Perspective/Awareness Medical/Clinical Knowledge	Participant Evaluation	Training enhances participants leadership competencies and attitudes towards working in interdisciplinary teams	2B	3
[25] **Weiss et al. 2020** **USA**	• SLP • Special education^a^	Interprofessional Experiential (clinical)	Medical/Clinical Knowledge	Participant Evaluation Intervention Evaluation Learning Assessment	Increases amongst all measures including: knowledge of transdisciplinary approach (TA), understanding and comfort with the other discipline, and higher confidence in using TA	3	4
[26] **Garavatti et al. 2018** **USA**	• Medicine • PT	Experiential (clinical) Interprofessional	Perspective/Awareness	Participant Evaluation	Students reported increased comfort in dealing with rehabilitation situations after attending the intervention	2A	3
[27] **Howell et al. 2012** **USA**	• OT • Psychology	Interprofessional Experiential (clinical)	Perspective/Awareness	Intervention Evaluation	Students were more prepared to represent their profession in an interprofessional team	1	1
[28] **Coret et al. 2018** **Canada**	• Medicine	Experiential (patient/family experiences)	Medical/Clinical Knowledge Perspective/Awareness	Learning Assessment Participant Evaluation Intervention Evaluation	Patient educators may help facilitate communication skills teaching amongst medical students	2B	3
[29] **Lewis et al. 2018** **Australia**	• SLP • OT	Experiential (clinical) Interprofessional	Medical/Clinical Knowledge	Intervention Evaluation	Students found the DVD role-playing interprofessional workshop to be a learning benefit	2A	2
[30] **Havercamp et al. 2016** **USA**	• Medicine	Theoretical (didactic) Experiential (patient/family experiences)	Medical/Clinical Knowledge Perspective/Awareness	Intervention Evaluation Participant Evaluation	Students reported improved knowledge, skills, confidence, and comfort in caring for patients with ASD	3	4
[31] **Watkins et al. 2016** **UK**	• Medicine	Experiential (clinical)	Perspective/Awareness Medical/Clinical Knowledge	Participant Evaluation	Students reported improvements in affect and understanding, as well as knowledge and skills	2B	4
[32] **Thomas et al. 2014** **UK**	• Medicine	Experiential (clinical) Theoretical (didactic)	Perspective/Awareness Medical/Clinical Knowledge	Participant Evaluation	Improvements in students’ perceived skills, comfort, and the type of clinical approach	3	4
[33] **Iacono et al. 2011** **Australia**	• Social work • OT • PT • Nursing • Other non-professional degrees^a^	Experiential (clinical) Interprofessional	Perspective/Awareness	Participant Evaluation Intervention Evaluation	No significant differences, although qualitative results show differences in perception and understanding	0	2
[34] **Feely et al. 2020** **Ireland**	• Social work	Theoretical (didactic) Experiential (clinical)	Perspective/Awareness Medical/Clinical Knowledge	Intervention Evaluation Participant evaluation	Students reported a positive experience with increased comfort and greater empathy	1	3
[35] **Marks et al. 2018** **Belgium**	• Dentistry	Experiential (clinical)	Medical/Clinical knowledge	Other	There were no significant changes in residents’ and caregivers' knowledge, behaviour, attitude, and self-efficacy on oral health	0	1
[36] **Watters et al. 2015** **USA**	• Dentistry	Experiential (clinical)	Medical/Clinical Knowledge Perspective/Awareness	Participant Evaluation	Improved self-efficacy and intent to treat patients with special needs	3	4
[37] **Harwood et al. 2014** **UK**	• Medicine	Theoretical (didactic) Experiential (patient/family experiences)	Medical/Clinical Knowledge Perspective/Awareness	Participant Evaluation	The online module has increased knowledge, skills, and reducing stigmatizing attitudes	4A	1
[38] **Jacobson et al. 2011** **USA**	• Medicine	Experiential (clinical)	Medical/Clinical Knowledge	Learning assessment	Students had more comfort with patients but showed no changes in their attitude or mental status examination performances	2A	1
[39] **Shields et al. 2014** **Australia**	• PT	Experiential (clinical)	Medical/Clinical Knowledge	Participant Evaluation	Students rated an improvement in their professional skills relating to implementing a Progressive Resistance Training programme	3	4
[40] **Karl et al. 2013** **USA**	• Medicine	Experiential (clinical) Immersive(Learning at a specialized medical facility)	Perspective/Awareness Medical/Clinical knowledge	Participant Evaluation	Improved comfort levels and increased awareness of attitudes that affect patient care	2B	4
[41] **Salama et al. 2020** **Saudi Arabia**	• Dentistry	Theoretical (didactic)	Medical/Clinical Knowledge	Participant Evaluation Learning Assessment	The intervention was effective in providing all levels of dental students with the basic instructive information to care for patients with IDD	2B	2
[42] **Taslibeyaz et al. 2017** **Turkey**	• Medicine	Theoretical (didactic) Experiential (clinical)	Medical/Clinical Knowledge	Learning Assessment	Increase in achievement scores for students in the interactive group	2B	4
[43] **Jones et al. 2015** **Canada**	• Medicine • Nursing • Clinical psych • OT • PT	Theoretical (didactic) Interprofessional Experiential (patient/family experiences)	Medical/Clinical Knowledge Perspective/Awareness	Participant Evaluation Learning Assessment	Significant differences in knowledge and skills following intervention Positive trend in students’ attitude changes following the intervention	2B	4
[44] **Tsilimingras et al. 2018** **USA**	• Medicine • Nursing • Psychology • Physician assistants • Post-graduate physicians^a^	Theoretical (didactic) Experiential (clinical) Interprofessional	Medical/Clinical Knowledge Perspective/Awareness	Participant Evaluation	Improvements in attitudes towards interprofessional clinical practice	2a	3
[45] **Phadraig et al. 2015** **Ireland**	• Dentistry	Theoretical (didactic) Experiential (workshops)	Perspective/Awareness	Participant Evaluation	No significant difference in student attitudes before and after intervention	0	1
[46] **Van Wieringen et al. 2015** **South Africa**	• Medicine	Theoretical (didactic) Experiential (patient/family experiences)	Other	Participant Evaluation	Positive differences found in quality and nature of IDD training on clinical rotations	2B	3
[47] **Hoang et al. 2023** **USA**	• Medicine	Theoretical (didactic) Experiential (clinical)	Medical/Clinical Knowledge	Participant Evaluation Learning Assessment	The virtual training sessions on behaviour analytic procedures increased students’ ability to apply such procedures in clinical roleplay with patients with neurodevelopmental disabilities	2B	4
[48] **Nash-Patel et al. 2022** **UK**	• Nursing	Experiential (patient/family experiences)	Perspective/Awareness	Participant Evaluation	The co-designed arts based relational learning programme was effective at reducing fears and anxieties between nurses and young patients with IDD	2A	2
[49] **Matteucci et al. 2023** **USA**	•Dentistry	Theoretical (didactic) Experiential (clinical)	Medical/Clinical Knowledge	Participant Evaluation Learning Assessment	Remote behaviour skills training for dental students and professionals was effective at encouraging providers to implement behaviour techniques in the care of patients with IDD	2B	3
[50] **Phadraig et al. 2022** **Ireland**	• Dentistry	Theoretical (didactic)	Perspective/Awareness	Participant Evaluation	A didactic training session led by an individual with autism promoted modest increases in openness towards caring for patients with autism	2A	3
[51] **Berger et al. 2023** **Canada**	• Medicine	Theoretical (didactic) Experiential (patient/family experiences)	Perspective/Awareness	Participant Evaluation	This curricular programme increased student confidence in interacting with patients with IDD but did not increase their sense of community inclusion	2A	4
[52] **Zencirci et al. ** **2022** **Turkey**	• Medicine	Theoretical (didactic) Experiential (clinical) Experiential (patient/family experiences)	Perspective/Awareness	Participant Evaluation	This mixed method training programme was effective in improving attitudes of senior medical students towards patients with IDD	2A	4
[53] **Jacob et al. ** **2022** **USA**	• Medicine	Theoretical (didactic) Experiential (clinical) Experiential (patient/family experiences)	Perspective/Awareness	Participant Evaluation	Medical students who participated in this programme reported improvements in comfort and confidence in interacting with patients with IDD and their families. However, families did not appear to trust physicians, with no significant changes after the program	2A	4
[54] **Lee et al. 2023** **USA**	• Nursing • Social work • Psychology • Recreation therapy^a^ • Exercise science^a^ • Public health^a^	Theoretical (didactic) Interprofessional	Perspective/Awareness	Participant Evaluation	This interprofessional program enhanced health professional students’ self-perceived competencies on the care of individuals with IDD	2A	3

^a^Not included in this study

**Table 2:** Review findings with a focus on intervention delivery

Author, Year of publication, COO	Training Speciality	Learner level	Instructor Type	Setting of intervention	Timeline of intervention
[23] **Keisling et al. 2017** **USA**	• Psychology • SLP • Audiology • Nutrition • Social work • Nursing	Unclear	Faculty members Patients, parents, or caregivers	Non-clinical	Longitudinal of longer than 3 months
[24] **Weber et al. ** **USA**	• Audiology • Genetic counselling • Nursing • OT • PT • Psychology • Social work • SLP • Other non- healthcare professional programs and/or post-graduate trainees^a^	Unclear	Faculty members	Non-clinical Clinical setting (unclear whether specialized or not)	Longitudinal of longer than 3 months
[25] **Weiss et al. 2020** **USA**	• SLP • Special education^a^	Unclear	Faculty members	Non-specialized clinical setting Non-clinical	Short-term less than 1 month
[26] **Garavatti et al. 2018** **USA**	• Medicine • PT	Medicine – 2^nd^ year PT – 3^rd^ year	Faculty members	Specialized clinical setting (community-based training facility for patients with DD)	Single session
[27] **Howell et al. 2012** **USA**	• OT • Psychology	1^st^ year and 2^nd^ year	Faculty members	Clinical setting (unclear whether specialized or not)	1-3 months
[28] **Coret et al. 2018** **Canada**	• Medicine	1^st^ year	Patients, parents, or caregivers Senior students	Non-clinical setting	Short term less than 1 month
[29] **Lewis et al. 2018** **Australia**	• SLP • OT	Unclear	Faculty members	Non-clinical	Single session
[30] **Havercamp et al. 2016** **USA**	• Medicine	3^rd^ year	Patients, parents, or caregivers Non-faculty professionals	Non-clinical	Single session
[31] **Watkins et al. 2016** **UK**	• Medicine	3^rd^ year	Patients, parents, or caregivers Non-faculty professionals	Non-clinical	Single session
[32] **Thomas et al. 2014** **UK**	• Medicine	4^th^ year	Faculty members Patients, parents, or caregivers Non-faculty professionals	Non- clinical	Single session
[33] **Iacono et al. 2011** **Australia**	• Social work • OT • PT • Nursing • Other non-professional degrees^a^	1^st^ year students 2^nd^ year tutors	Patients, parents, or caregivers Senior students	Non- clinical	Single session
[34] **Feely et al. 2020** **Ireland**	• Social work	3^rd^ year	Faculty members Patients, parents, or caregivers	Non-clinical	Longitudinal of longer than 3 months
[35] **Marks et al. 2018** **Belgium**	• Dentistry	4^th^ year	Unclear	Specialized clinical setting	1–3 months
[36] **Watters et al. 2015** **USA**	• Dentistry	4^th^ year	Faculty members	Non-clinical Specialized clinical setting	1–3 months
[37] **Harwood et al. 2014** **UK**	• Medicine	4^th^ year	Faculty members Patients, parents, or caregivers	Non- clinical	Unclear
[38] **Jacobson et al. 2011** **USA**	• Medicine	3^rd^ year	Faculty members	Specialized clinical setting	Short term less than 1 month
[39] **Shields et al. 2014** **Australia**	• PT	Various years	Unclear	Non- specialized clinical setting	1–3 months
[40] **Karl et al. 2013** **USA**	• Medicine	3^rd^ year	Faculty members Non-faculty professionals	Specialized clinical setting	Single session
[41] **Salama et al. 2020** **Saudi Arabia**	• Dentistry	Various years	Unclear	Non-clinical	Single session
[42] **Taslibeyaz et al. 2017** **Turkey**	• Medicine	Various years	Unclear	Non-clinical	Single session
[43] **Jones et al. 2015** **Canada**	• Medicine • Nursing • Clinical psych • OT • PT	Various years	Faculty members Patients, parents, or caregivers	Non- clinical	Single session
[44] **Tsilimingras 2018** **USA**	• Medicine • Nursing • Psychology • Physician assistants • Post-graduate physicians^a^	Various years	Faculty members	Non-clinical	Single session
[45] **Phadraig et al. 2015** **Ireland**	• Dentistry	3^rd^ year	Faculty members Patients, parents, or caregivers	Specialized clinical setting Non-specialized clinical setting Non-clinical	1–3 months
[46] **Van Wieringen et al. 2015** **South Africa**	• Medicine	2^nd^ year and 4^th^ year	Faculty members	Unclear	Longitudinal of longer than 3 months
[47] **Hoang et al. 2023** **USA**	• Medicine	2^nd^, 3^rd^, 4^th^ year	Unclear	Non-specialized clinical setting Non-clinical	1–3 months
[48] **Nash-Patel et al. 2022** **UK**	• Nursing	2^nd^ and 3^rd^ year	Faculty members Patients, parents, or caregivers Non-faculty professionals	Non-clinical	1–3 months
[49] **Matteucci et al. 2023** **USA**	• Dentistry	2^nd^ and 4^th^ year	Faculty members Patients, parents, or caregivers	Specialized clinical setting	Short term less than 1 month
[50] **Phadraig et al. 2022** **Ireland**	• Dentistry • Dental hygiene	Dentistry—3^rd^ year dental Dental hygiene and Nursing—2^nd^ year	Patients, parents, or caregivers Faculty members Non-faculty professionals	Non-clinical	Single session
[51] **Berger et al. 2023** **Canada**	• Medicine	1^st^ year students	Faculty members Patients, parents, or caregivers Non-faculty professionals	Non-clinical	1–3 months
[52] **Zencirci et al. 2022** **Turkey**	• Medicine	Unclear	Faculty members Patients, parents, or caregivers	Non-clinical Specialized clinical setting	Short-term less than 1 month
[53] **Jacob et al. 2022** **USA**	• Medicine	1^st^ and 2^nd^ years	Faculty members Patients, parents, or caregivers	Non- specialized clinical setting	1–3 months
[54] **Lee et al. 2023** **USA**	• Nursing • Social work • Psychology • Recreation therapy^a^ • Exercise science^a^ • Public health^a^	Unclear	Faculty members Patients, parents, or caregivers Non-faculty professionals	Non-clinical	Longitudinal of longer than 3 months

^a^Not included in this study

### Study characteristics

Table 1 summarizes study characteristics for the 32 included studies. Starting from the largest proportion of studies, 16% (5/32) of the studies were published in 2018 [26, 28, 29, 35, 44], and 13% (4/32) of the studies were published in 2015 [36, 43, 45, 46], 2022 [48, 50, 52, 53], and 2023 [47, 49, 51, 54], each. Years 2014 [32, 37, 39] and 2020 [25, 34, 41] made up 9% (3/32) of the studies, each, and 2011 [33, 38], 2016 [30, 31] and 2017 [23, 42] made up 6% (2/32) of the studies, each. Finally, 2012 [27], 2013 [40], and 2021 [24] made up 3% (1/32) of the studies, each.

The majority of the studies were conducted in the United States of America (44%, 14/32) [23–27, 30, 36, 38, 40, 44, 47, 49, 53, 54], followed by the UK (13%, 14/32) [31, 32, 37, 48], Australia (9%, 3/32) [29, 33, 39], Canada (9%, 3/32) [28, 43, 51], Ireland (9%, 3/32) [34, 45, 50], Turkey (6%, 2/32) [42, 52], Belgium (3%, 1/32) [35], Saudi Arabia (3%, 1/32) [41], and South Africa (3%, 1/32) [46].

With regards to trainee demographics, most of the studies were specifically targeted towards medical students (50%, 16/32) [26, 28, 30–32, 37, 38, 40, 42–44, 46, 47, 51–53]. Following medical students, were nursing (25%, 8/32) [23, 24, 33, 43, 44, 48, 50, 54], dentistry (19%, 6/32) [35, 36, 41, 45, 49, 50], psychology (19%, 6/32) [23, 24, 27, 43, 44, 54], physiotherapy (16%, 5/32) [24, 26, 33, 39, 43], occupational therapy (16%, 5/32) [24, 27, 29, 33, 43], social work (16%, 5/32) [23, 24, 33, 34, 54], and speech language pathology (13%, 4/32) [23–25, 29] students. Other specialities included in IDD interventions were, audiology (6%, 2/32) [23, 24], nutrition (3%, 1/32) [23], physician assistant (3%, 1/32) [44], dental hygiene (3%, 1/32) [50], and genetic counselling (3%, 1/32) [24].

As for trainees’ year in their respective programs, the results were varied with the most studies including 3^rd^ years (31%, 10/32) [26, 30, 31, 34, 38, 40, 45, 47, 48, 50], followed by 2^nd^ years (28%, 9/32) [26, 27, 33, 46–50, 53], 4^th^ years (22%, 7/32) [32, 35–37, 46, 47, 49], and 1^st^ years (16%, 5/32) [27, 28, 33, 51, 53]. However, almost half of the studies were unclear with regards to learner level (19%, 6/32) [23–25, 29, 52, 54] or included trainees of all years (16%, 5/32) [39, 41–44].

### Curriculum characteristics

Many of the interventions included faculty members (72%, 23/32) [23–27, 29, 32, 34, 36–38, 40, 43–46, 48–54] and/or patients, parents, or caregivers (53%, 17/32) [23, 28, 30–34, 37, 43, 45, 48–54] as instructors. Moreover, some studies utilized the expertise of non-faculty professionals as instructors (25%, 8/32) [30–32, 40, 48, 50, 51, 54]. Interestingly, a few studies capitalized on the past experiences of previous trainees and/or senior students using them as instructors (6%, 2/32) [28, 33]. Although, for 16% (5/32) of the studies, the instructor type was categorized as unclear [35, 39, 41, 42, 47].

The majority of interventions were single sessions (38%, 12/32) [26, 29–33, 40–44, 50]. On the other hand, there were several studies that were longitudinal of longer than 3 months (16%, 5/32) [23, 24, 34, 46, 54], however some of these studies were non-continuous, and often had varying amounts of time between sessions. Additionally, a significant number of studies were 1–3 months in length (28%, 9/32) [27, 35, 36, 39, 45, 47, 48, 51, 53], and the minority of studies were short-term of less than 1 month (16%, 5/32) [25, 28, 38, 49, 52].

As for the setting of intervention, the majority included non-clinical settings (75%, 24/32) [23–25, 28–34, 36, 37, 41–45, 47–52, 54], followed by specialized clinical settings (22%, 7/32) [26, 35, 38, 40, 45, 49, 52], and non-specialized clinical settings (16%, 5/32) [25, 39, 45, 47, 53]. As well, some of the settings were classified as clinical but lacked clarity on whether the setting was a specialized centre or not (6%, 2/32) [24, 27]. Finally, for 3% (1/32) of the studies, the setting of intervention was unclear [46].

### Pedagogical approach

Most of the studies used experiential approaches to teaching (88%, 28/32). Experiential activities typically included a clinical experience (63%, 20/32) [24–27, 29, 31–36, 38–40, 42, 44, 47, 49, 52, 53], which were defined as any intervention that recreated or involved a clinical encounter, some examples include simulations with standardized patients or role playing (6%, 2/14) [29, 31]. Other forms of experiential teaching took the form of narrative patient/parents/caregiver experiences (31%, 10/32) [23, 28, 30, 37, 43, 46, 48, 51–53] and workshops (3%, 1/32) [45]. As well, many of the studies utilized a theoretical approach to teaching (59%, 19/32) [23, 24, 30, 32, 34, 37, 41–47, 49–54], often in the form of didactic lectures. However, some studies utilized case studies, educational DVDs, and interactive virtual scenarios to teach theory. In addition, while still didactic, some studies utilized patients/parents/caregivers as instructors and curriculum developers. Finally, a large proportion of studies utilized interprofessional education (35%, 11/32) [23–27, 29, 33, 43, 44, 50, 54]. Interprofessional methods were always found in addition to other approaches to learning such as experiential and/or theoretical.

### Educational outcomes

A variety of evaluation methods were used to assess intervention outcomes. Participant evaluations of their own learning were overwhelmingly used (84%, 27/32) [23–26, 28, 30–34, 36, 37, 39–41, 43–54]. Often, participant evaluations took the form of pre and post intervention surveys, whereby participants were compared to their pre-intervention scores. Evaluations were also done in the form of learning assessments, where acquired knowledge was tested (28%, 9/32) [25, 28, 38, 41, 42, 42, 43, 47, 49]. Some studies chose to evaluate the intervention itself through participant surveys rating intervention design and effectiveness (22%, 7/32) [25, 27–30, 33, 34]. One of the studies had no evaluation method for learners, as it was a community service-learning experience that focused on community outcomes [35].

As for study outcomes, the Kirkpatrick model was applied to evaluate the outcomes of the educational interventions. Our review produced a mean and median of 2.16 and 2.5, respectively (if 2A = 2.0 and 2B = 2.5). In order of scoring, 9% (3/32) [33, 33, 45] of the studies were graded level 0 due to lack of change demonstrated, 6% (2/32) [27, 34] were graded level 1 indicating only a reaction to the learning experience, 31% (10/32) [26, 29, 38, 44, 48, 50–54] were graded level 2A indicating a change in attitude, 31% (10/32) [24, 28, 31, 40–43, 46, 47, 49] were graded level 2B indicating a modification of knowledge or skills, 19% (6/32) [23, 25, 30, 32, 36, 39] were graded level 3 indicating a change in behaviour, and 3% (1/32) [37] of the studies were graded level 4A indicating a change in the system/organization practice. No papers were graded level 4B as no significant improvements in student performance as a direct result of the education were seen.

Our BEME evidence-based scoring system review produced a mean and median of 3 and 3, respectively. We graded 16% (5/32) [27, 35, 37, 38, 45] of papers as a grade 1 – no clear conclusions can be deduced, 13% (4/32) [29, 33, 41, 48] of papers as a grade 2 – ambiguous results, although appearance of a trend, 28% (9/32) [24, 26, 28, 34, 44, 46, 49, 50, 54] of papers as a grade 3 – conclusions can probably be based on the findings, and 44% (14/32) [23, 25, 30–32, 36, 39, 40, 42, 43, 47, 51–53] papers as grade 4 – results are clear and very likely to be true. No papers were graded as 5 – results are unequivocal due to generally small samples and large reliance on questionnaires with no longitudinal evaluations.

## Discussion

Through this systematic review, we aimed to summarize the literature surrounding pre-graduate healthcare professional training in IDD. Our analysis has brought forward several points of importance in IDD curriculum design. In particular, we saw that many of the highest BEME scores [23, 25, 30, 32, 40, 43, 52, 53] and Kirkpatrick outcomes [23, 25, 30, 32, 37] were interventions that included multiple pedagogical methods. This is corroborated by previous research suggesting that multimodal approaches to educational programmes have improved educational outcomes [4].

We found that the majority of interventions were a single session intervention (38%, 12/32) [26, 29–33, 40–44, 50]. At the same time, there were several studies that were longitudinal of longer than 3 months (16%, 5/32), although only few were continuous over the time of intervention. Notably, many of the interventions seemed to be pilot studies instead of integrated components of the pre-graduate curriculum. While these pilot studies displayed relatively similar BEME scores and Kirkpatrick levels compared to the long-term studies, the latter often gave importance to leadership and advocacy related competencies. This suggests an emphasis on developing leadership and advocacy as a response to the needs of an under-served and marginalized population. Similar to this, the study by Mullin et al. highlights the importance of equity, diversity and inclusion (EDI) in health leadership as a means to dismantle the oppression of a marginalized population through system level changes [55]. Thus, principles of leadership and advocacy embedded in EDI, and more specifically IDD education, may be essential to addressing the needs of the IDD population through a top-down approach. As well, long-term studies were more likely to involve a curriculum review with the potential for curriculum improvement when compared to pilot studies [37, 46]. Therefore, a shift towards ongoing, continuous curricula may better support the development of our future healthcare leaders and advocates.

Interestingly, an interprofessional approach to education was found amongst several studies. The mean BEME scores and Kirkpatrick levels for these interventions were 2.9 and 2, respectively. While these scores reflect some gains in knowledge and/or perspective, this was less than expected given the promising literature on interprofessional education and improvements in educational outcomes [56–60]. A possible reason for these scores could be the study design used, with more emphasis on team-based dynamics as opposed to individual knowledge attainment. This finding was highlighted in the study done by Keshmiri et al. where an interprofessional education session with medical students, nurses, and medical residents found some improvements in participants’ self-efficacy, but even higher improvements in interprofessional performance [57]. Similar results were found in the study done by Hamilton et al. where they found gains in professional skills following an interprofessional education session event with nursing and medical students were better retained 6 months later compared to gains in clinical and technical skills [61]. These findings suggest that interprofessional education in IDD training produces individual benefits but more substantially benefits team dynamics amongst healthcare professionals.

Furthermore, it is essential to analyze our findings through a critical disability lens to ensure a comprehensive and equitable interpretation. Critical disability studies view disability as both a lived reality in which the experiences of people with disabilities are central to interpreting their place in the world, and as a social and political definition based on societal power relations [62]. Inclusion of people’s lived experience is important but particularly valuable when framed by them, and when learning about their lives considers the systemic barriers they face, as opposed to a medicalized view of their illnesses/impairments. Many of the studies in this review have integrated patient and family experience in the pedagogy (53%, 17/32). However, studies seldom adopted a critical disability lens, which would have contextualized patients' experiences of disparities within broader social systems. Further, co-production and co-delivery in pedagogical approaches can help bring transformative changes in learners, and consequently in the health systems supporting care of persons with IDD. Such approaches have been considered in the past to understand how coproduction can support humanistic education and transformative learning [63]. Curriculum developers can embrace a critical disability lens in IDD curriculum design to drive system changes and improve health equity. Strategies such as application of a health equity and inclusion framework to support equity and inclusion in planning, development and implementation of IDD curricula, can be considered [64].

The findings of our study were limited by the inclusion of only English publications, despite an international scope. As well, we excluded studies that did not describe a clear intervention. For instance, we excluded a review of Australian medical schools’ IDD education over 20 years as it focused on summarizing the current curriculum to inform revision, rather than a discussion of intervention characteristics or educational outcomes [8]. Moreover, it is likely that IDD interventions well integrated into pre-graduate curricula may not have been published, and so were not captured in this review. Finally, our review included only 1 reviewer which may have introduced bias during the selection and analysis process. Despite these limitations, we believe the findings strongly highlight the need for formal pre-graduate IDD education.

## Conclusions

In conclusion, this review of IDD curricula in international pre-graduate health professional.education has provided an overview of published interventions and highlighted several trends. First, the literature in this field supports the use of multimodal approaches to achieve greater educational outcomes. Program developers can consider the use of multiple pedagogical methods in IDD curricula. Second, many interventions were single-session, pilot studies. There is a need for longitudinal learning opportunities and consistency through integration into formal curricula, which should also be formally evaluated. Third, interprofessional components to education are increasingly being used. Future studies can integrate team competencies and its evaluation along with IDD self-efficacy outcomes. Finally, while interventions frequently involved patients and caregivers in their design and implementation, these experiences were seldom situated within the larger systemic disparities faced by patients with IDD. To strengthen this approach, future studies could adopt a critical disability lens to gain deeper insights into patients' lived realities and to advocate for systemic change. In summary, there is an increased need for formal, effective IDD education for healthcare professionals. It is especially important that this education be directed at the level of pre-graduate training to equip health care professionals with the skills and attitudes to care for those with IDD before entering the workforce.

## Supplementary Information

**Additional file 1:** **Table S1.** Summary of reviewed literature on IDD teaching in pre-graduate health professional training.

## Data Availability

All data generated or analysed during this study are included in this published article and its supplementary information files.

## Abbreviations

BEME: Best Evidence Medical Education Guide
COO: Country of Origin
EDI: Equity, Diversity, and Inclusion
IDD: Intellectual and Developmental Disabilities
OT: Occupational Therapy
PRISMA: Preferred Reporting Items for Systematic Reviews and Meta-Analysis
PT: Physiotherapy/ Physical Therapy
