# Supplementary material for: A systematic review of intellectual and developmental disability curriculum in international pre-graduate health professional education
Source: BMC Med Educ. 2023 May 11;23:329. doi: 10.1186/s12909-023-04259-4 (PMC10176941; doi:10.1186/s12909-023-04259-4)
Supplement: Supplementary file 1 — Additional file 1: Table S1. Summary of reviewed literature on IDD teaching in pre-graduate health professional training. [file 12909_2023_4259_MOESM1_ESM.docx]

Table S1. Summary of reviewed literature on IDD teaching in pre-graduate health professional training.

| 1. **Year of Publication** | **(%, n)** | **References** |
| --- | --- | --- |
| - 2011 | (6%, 2) | [33,38] |
| - 2012 | (3%, 1) | [27] |
| - 2013 | (3%, 1) | [40] |
| - 2014 | (9%, 3) | [32,37,39] |
| - 2015 | (13%, 4) | [36,43,45,46] |
| - 2016 | (6%, 2) | [30,31] |
| - 2017 | (6%, 2) | [23,42] |
| - 2018 | (16%, 5) | [26,28,29,35,44] |
| - 2020 | (9%, 3) | [25,34,41] |
| - 2021 | (3%,1) | [24] |
| - 2022 | (13%, 4) | [48,50,52,53] |
| - 2023 | (13%, 4) | [47,49,51,54] |
|  |  |  |
| 1. **Country of Origin** |  |  |
| - USA | (44%, 14) | [23–27,30,36,38,40,44,47,49,53,54] |
| - UK | (13%, 14) | [31,32,37,48] |
| - Australia | (9%, 3) | [29,33,55] |
| - Canada | (9%, 3) | [28,43,51] |
| - Ireland | (9%, 3) | [34,45,50] |
| - Turkey | (6%, 2) | [42,52] |
| - Belgium | (3%, 1) | [35] |
| - Saudi Arabia | (3%, 1) | [41] |
| - South Africa | (3%, 1) | [46] |
|  |  |  |
| 1. **Specialities Included** |  |  |
| - Medicine | (50%, 16) | [26,28,30–32,37,38,40,42–44,46,47,51–53] |
| - Nursing | (25%, 8) | [23,24,33,43,44,48,50,54] |
| - Dentistry | (19%, 6) | [35,36,41,45,49,50] |
| - Psychology | (19%, 6) | [23,24,27,43,44,54] |
| - Social work | (16%, 5) | [23,24,33,34,54] |
| - Occupational Therapy (OT) | 16%, 5) | [24,27,29,33,43] |
| - Physiotherapy (PT) | (16%, 5) | [24,26,33,43,55] |
| - Speech language pathology (SLP) | (13%, 4) | [23–25,29] |
| - Audiology | (6%, 2) | [23,24] |
| - Genetic Counselling | (3%, 1) | [50] |
| - Physician Assistant | (3%, 1) | [44] |
| - Nutrition | (3%, 1) | [23] |
| - Dental Hygiene | (3%, 1) | [24] |
|  |  |  |
| 1. **Learner Level of Participants** |  |  |
| - Unclear | 19%, 6) | [23–25,29,52,54] |
| - Various years (all years of program included) | (16%, 5) | [41–44,55] |
| - 1^st^ year | (16%, 5) | [27,28,33,51,53] |
| - 2^nd^ year | (28%, 9) | [26,27,33,46–50,53] |
| - 3^rd^ year | (31%, 10) | [26,30,31,34,38,40,45,47,48,50] |
| - 4^th^ year | (22%, 7) | [32,35–37,46,47,49] |
|  |  |  |
| 1. **Instructor Type** |  |  |
| - Faculty members | (72%, 23) | [23–27,29,32,34,36–38,40,43–46,48–54] |
| - Patients, parents, or caregivers | (53%, 17) | [23,28,30–34,37,43,45,48–54] |
| - Unclear | 16% (5) | [35,41,42,47,55] |
| - Non-faculty professionals | (25%, 8) | [30–32,40,48,50,51,54] |
| - Senior students | (6%, 2) | [28,33] |
|  |  |  |
| 1. **Setting of intervention** |  |  |
| - Non-clinical | (75%, 24) | [23–25,28–34,36,37,41–45,47–52,54] |
| - Specialized clinical setting | (22%, 7) | [26,35,38,40,45,49,52] |
| - Non-specialized clinical setting | (16%, 5) | [25,39,45,47,53] |
| - Clinical setting (unclear if specialized) | (6%, 2) | [24,27] |
| - Unclear | (3% (1) | [46] |
|  |  |  |
| 1. **Timeline of Intervention** |  |  |
| - Single session | (38%, 12) | [26,29–33,40–44,50] |
| - 1-3 months | (28%, 9) | [27,35,36,45,47,48,51,53,55] |
| - Longitudinal of longer than 3 months | (16%, 5) | [23,24,34,46,54] |
| - Short-term less than 1 month | (16%, 5) | [25,28,38,49,52] |
| - Unclear | (3%, 1) | [37] |
|  |  |  |
| 1. **Pedagogical Methodology** |  |  |
| - Experiential (Patient/family experiences) | (31%, 10) | [23,28,30,37,43,46,48,51–53] |
| - Experiential (Clinical) | (63%, 20) | [24–27,29,31–36,38–40,42,44,47,49,52,53] |
| - Experiential (Workshops) | (3%, 1) | [45] |
| - Theoretical | (59%, 19) | [23,24,30,32,34,37,41–47,49–54] |
| - Interprofessional | (35%, 11) | [23–27,29,33,43,44,50,54] |
|  |  |  |
| 1. **Focus of Content** |  |  |
| - Medical/clinical knowledge | (63%, 20) | [24,25,28–32,34–44,47,49] |
| - Perspective/awareness | (69%, 22) | [23,24,26–28,30–34,36,37,40,43–45,48,50–54] |
| - Other | (3%, 1) | [46] |
|  |  |  |
| 1. **Evaluation Methodology** |  |  |
| - Participant evaluation | (84%, 27) | [23–26,28,30–34,36,37,39–41,43–54] |
| - Learning assessment | (28%, 9) | [25,28,38,41,42,42,43,47,49] |
| - Intervention evaluation | (22%, 7) | [25,27–30,33,34] |
| - Unclear | (3%, 1) | [35] |
|  |  |  |
| 1. **Kirkpatrick Level** |  |  |
| - Level 0 | (9%, 3) | [33,33,45] |
| - Level 1 | (6%, 2) | [27,34] |
| - Level 2A | (31%, 10) | [26,29,38,44,48,50–54] |
| - Level 2B | (31%, 10) | [24,28,31,40–43,46,47,49] |
| - Level 3 | (19%, 6) | [23,25,30,32,36,55] |
| - Level 4A | (3% 1) | [37] |
| - Level 4B | (0%, 0) |  |
|  |  |  |
| 1. **BEME Scores** |  |  |
| - Grade 1 | (16%, 5) | [27,35,37,38,45] |
| - Grade 2 | (13%, 4) | [29,33,41,48] |
| - Grade 3 | (28%, 9) | [24,26,28,34,44,46,49,50,54] |
| - Grade 4 | (44%, 14) | [23,25,30–32,36,39,40,42,43,47,51–53] |
| - Grade 5 | (0%, 0) |  |
